# Implementation and Effectiveness of a Bar Code–Based Transfusion Management System for Transfusion Safety in a Tertiary Hospital: Retrospective Quality Improvement Study

**DOI:** 10.2196/14192

**Published:** 2019-08-26

**Authors:** Shin-Shang Chou, Ying-Ju Chen, Yu-Te Shen, Hsiu-Fang Yen, Shu-Chen Kuo

**Affiliations:** 1 Department of Nursing Taipei Veterans General Hospital Taipei City Taiwan; 2 School of Nursing National Yang-Ming University Taipei Taiwan; 3 School of Nursing Taipei Medical University Taipei Taiwan; 4 Section of Transfusion Medicine Department of Medicine Taipei Veterans General Hospital Taipei Taiwan; 5 Department of Information Management Taipei Veterans General Hospital Taipei Taiwan

**Keywords:** blood transfusion safety, barcode technology, quality improvement

## Abstract

**Background:**

Large-scale and long-term studies are not sufficient to determine the efficiency that IT solutions can bring to transfusion safety.

**Objective:**

This quality-improvement report describes our continuous efforts to implement and upgrade a bar code–based transfusion management (BCTM) system since 2011 and examines its effectiveness and sustainability in reducing blood transfusion errors, in a 3000-bed tertiary hospital, where more than 60,000 prescriptions of blood transfusion are covered by 2500 nurses each year.

**Methods:**

The BCTM system uses barcodes for patient identification, onsite labeling, and blood product verification, through wireless connection to the hospital information systems. Plan-Do-Study-Act (PDSA) cycles were used to improve the process. Process maps before and after implementation of the BCTM system in 2011 were drawn to highlight the changes. The numbers of incorrect labeling or wrong blood in tube incidents that occurred quarterly were plotted on a run chart to monitor the quality changes of each intervention introduced. The annual occurrences of error events from 2011 to 2017 were compared with the mean occurrence of 2008-2010 to determine whether implementation of the BCTM system could effectively reduce the number of errors in 2016 and whether this reduction could persist in 2017.

**Results:**

The error rate decreased from 0.03% in 2008-2010 to 0.002% in 2016 (*P*<.001) and 0.001% in 2017 (*P*<.001) after implementation of the BTCM system. Only one incorrect labeling incident was noted among the 68,324 samples for blood typing, and no incorrect transfusions occurred among 67,423 transfusion orders in 2017.

**Conclusions:**

This report demonstrates that continuous efforts to upgrade the existing process is critical to reduce errors in transfusion therapy, with support from information technology.

## Introduction

Blood transfusion is a complex multistep process that includes confirming the doctors’ prescriptions, sampling and testing the patients’ blood, preparing and storing the blood components, and delivering the needed components to patients. These steps, involving members of several different professional groups, have several hotspots for errors that need to be checked to protect transfusion safety [1]. In the 2010 World Health Organization’s guidelines for National Health Authorities and Hospital Management for Clinical Transfusion Process and Patient Safety, the need for the implementation of standardized procedures throughout the clinical transfusion process, including patient identification, blood administration, and patient monitoring was emphasized [2].

Despite many efforts to prevent transfusion errors, there is room for improvement. In the 2017 annual report of Serious Hazards of Transfusion (SHOT), an independent, professionally led hemovigilance scheme of the United Kingdom, clearly states that “...Many such errors could be attributed to system faults and others to what we now call ‘human factors’...we must design our practices and systems to minimise the impact...” SHOT recommends that “All available information technology [IT] systems to support transfusion practice should be considered and these systems implemented to their full functionality...” for the management and the transfusion teams of hospitals [3].

Although the application of new technology could simplify the complexity of routine procedures and an end-to-end electronic system could help further improve transfusion safety [4-7], the drive of using IT technologies to improve transfusion-related errors is lacking worldwide. Obstacles to the deployment of new technology include resistance to change, confusion regarding the best technology, and uncertainty regarding the return on investment [8]. Possible reasons for resistance to implementing technology are the multifaceted cost of technology, underestimation of errors, viewing technology as new and confusing, and even mistakenly assuming that errors are simply a “bad nurse” issue [9]. Large-scale and long-term studies are also not sufficient to support the efficiency that IT solutions can bring to transfusion safety. Recent reports from the transfusion error surveillance system of Canada [9] and the Q-Probes Study by the College of American Pathologists have found that the use of bar coding was not associated with lower mislabeling or wrong blood in tube (WBIT) rates [10].

The objectives of this paper are to describe our efforts since 2011 to develop a bar code–based transfusion management system (BCTM) and to test if full implementation of BCTM in our hospital, a 3000-bed tertiary care hospital, could result in a significant reduction in transfusion errors in 2016 and whether this reduction could persist in 2017.

## Methods

### Design

This is a retrospective study. The format of this quality improvement report follows the Standards for QUality Improvement Reporting Excellence (SQUIRE 2.0) guidelines [11].

### Setting

The study hospital has approximately 2500 first-line nurses to deliver more than 5000 blood transfusion therapies each month. Based on the International Business Machines (IBM) framework built in 1982, the hospital information system (HIS) consists of many subsystems such as the computerized physician order entry (CPOE) system, the laboratory information system (LIS), the pharmacy information system, and the nursing information system (NIS). Although each subsystem has been developed and evolved over time to serve particular needs, these subsystems are all linked within the HIS. The unique patient identification (ID) number is the key to retrieve relevant information for a particular patient from the HIS. With the completion of the whole-hospital wireless system and the deployment of mobile nursing carts, which are equipped with an industrial computer wirelessly linked to the HIS, in 2009, it became possible for nurses to retrieve and verify relevant information at bedside for patient-centered services. The bar code medication administration (BCMA) system, deployed in 2010, was the first system in the study hospital to use barcode scanning of the patient’s wristband for patient ID.

Inspired by Murphy’s [12] work on the electronic control of blood transfusions in Oxford in 2008, Askeland’s [13] barcode–based tracking system used to improve transfusion safety in Iowa [13], and the lessons learned from our BCMA, the nursing department of the study hospital assembled a BCTM project team in June 2010 to improve transfusion processes with an objective to reduce the near-miss rate to fewer than three incidents per quarter (ie, 1 per 5000 orders monthly). The BCTM project team consists of nurses, information technologists, and Blood Centre technicians and is led by the director of quality management of the nursing department. The team uses Plan-Do-Study-Act cycles and the model for improvement developed by the associates in process improvement as the framework to redesign the process [14].

The project team first reviewed the root causes of the 41 wrong labeling incidents that occurred in 2008-2010 and found that 17 incidents (41%) were caused by staff being interrupted by other urgent issues and 6 mistakes (14%) were related to complicated sticker and paper requisition forms (Table 1). Nine incidents (22%) were the result of staff being unfamiliar with the procedure. In 4 cases (10%), staff were unable to perform two-person verification due to a lack of staff members and in 5 instances (12%), there were deviations from the standard operating procedures.

These findings suggest the need for a close working environment to avoid interruptions, less complicated sticker/paper forms, and streamlined procedures to improve compliance.

The BCTM project team also reviewed the process of blood sampling (Table 2). With inputs from first-line nurses and field surveys of the acceptance of the BCMA, the project team found the following: the batch preparation of sampling tubes at the nursing station by night shift nurses with multiple requests was handled simultaneously using preprinted stickers of the patient’s name from chart boards for labeling, batch preparation might have caused confusion and mislabeling, and it relied too much on paper requisition forms printed from the HIS terminal.

Under daily routine situations, to avoid repeat blood drawing from a patient, all types of blood samples for each patient are collected together in the early morning. Under emergency conditions, the nurse performs the blood sampling immediately.

**Table 1:** The causes of errors of labeling in 2008-2010 (N=41).

Causes of errors	Value, n (%)
Interrupted by other urgent issues	17 (41)
Staff unfamiliar with the procedure	9 (22)
Staff deviated from the standard operating procedure	5 (12)
Understaffing to perform double check at bedside	4 (10)
Patient’s sticker misplaced	3 (7)
Wrong stickers or requisition on sample tube/bag	3 (7)

**Table 2:** Process changes in blood sampling for grouping.

Before BCTM^a^	After BCTM
HIS^b^ terminal prints out order for blood typing at the station Ward clerk notifies nurse providing care Nurse confirms the order from medical chart and puts the standing orders into a box for blood sampling the next morning Evening shift nurse prepares tubes for blood typing and labels the tube with the preprinted ID^c^ sticker at the station	HIS terminal prints out order for blood typing at the station Ward clerk notifies nurse providing care Nurse confirms the order from a mobile unit
Early morning shift nurse brings the prelabeled tubes and paper requisition forms to bedside Talks to the patient of the upcoming procedures Performs two-person verification of patient identification and order by reading out and repeating the necessary information on the patient’s ID and requisition forms Draws blood for typing and fills into the prelabeled tube Two nurses double sign the requisition form Wraps the filled prelabeled tube with the requisition form Returns wrapped tubes to station The ward clerk writes down the requisition number of all tubes on a list for sample tracking The porter signs the list and sends the samples to the blood bank	Early morning shift nurse moves to bedside with a phlebotomy cart Talks to the patient of the upcoming procedures Scans patient’s wristband for patient ID and verifies orders through the BCTM system Draws blood for typing and fills into the selected tube After the second staff verifies data through BCTM, a sticker containing necessary information and barcodes is printed out for on-site labeling Wraps the labeled tube with paper requisition form (discontinued after June 2013) Returns the labeled tube to the station The porter scans each sample’s barcode and sends the samples to the blood bank

^a^BCTM: Bar Code based Transfusion Management.

^b^HIS: hospital information system.

^c^ID: identification.

### Interventions

#### Using Barcoding for Patient Identification and Information Linkage

Based on the abovementioned review, the BCTM team adopted the scanning of wristband barcodes of patient ID for timely verification and documentation on each transaction of transfusion therapy from all relevant subsystems (ie, CPOE, LIS, and NIS) of HIS and proposed the following three major changes: (1) label sample tubes at bedside, (2) redesign the end-to-end tracking of transfusion therapy, and (3) provide step-by-step reminders. The standard procedures for blood sampling and blood product administration were also updated accordingly (Table 3).

**Table 3:** Process changes in blood product administration.

Before BCTM^a^	After BCTM
Blood product arrives at the nursing station Ward clerk notifies the caring nurse Nurse checks the information of the blood product and the standing prescription of transfusion from medical chart of the patient	Blood product arrives at the Nursing Station Ward clerk notifies the caring nurse Nurse scans the barcode on the blood bag to verify the transfusion prescription and the right blood product in BCTM
Nurse brings the blood product and medical chart/paper order to the bedside Talks to the patient of the upcoming procedures Performs two-person verification by reading out and repeating the information of patient identification, blood bag content, and the prescription of transfusion therapy Starts transfusion and monitoring Records patient’s responses to transfusion into the NIS^c^ Writes on the paper form of transfusion reaction record of patient’s response Returns transfusion record to the station to confirm the completion of the transfusion Ward clerk sends the paper record to the blood bank for tracking	Nurse brings the blood product to the patient with a nursing cart Talks to the patient of the coming procedures Scans patient’s wristband ID^b^ and the barcode on the blood bag to verify the order in BCTM A second staff member repeats the abovementioned processes Starts transfusion and monitoring Records patient’s responses to transfusion into the NIS Generates transfusion reaction record from NIS Confirms the completion of transfusion through BCTM for electronic tracking

^a^BCTM: Bar Code based Transfusion Management.

^b^ID: identification.

^c^NIS: nursing information system.

#### Labeling Sample Tubes at Bedside

**Figure 1:**
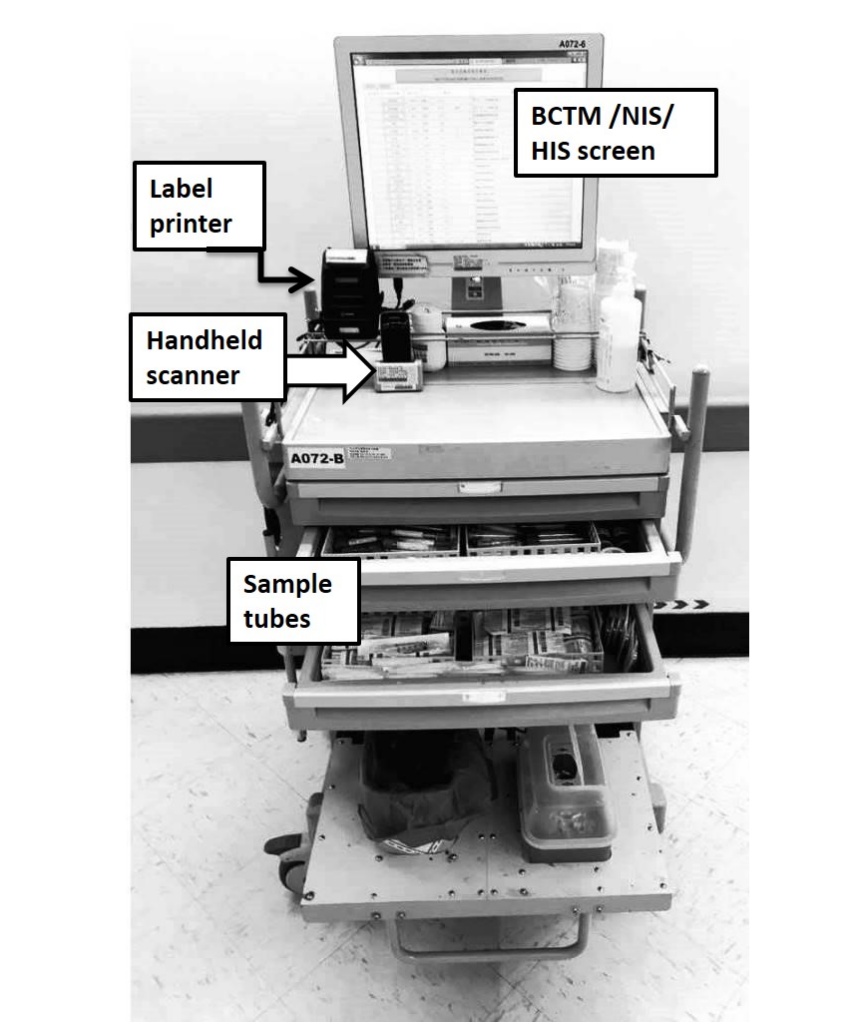
Layout of the phlebotomy cart. BCTM: bar code–based transfusion management; ID: identification; HIS: hospital information system; NIS: Nursing Information System.

**Figure 2:**
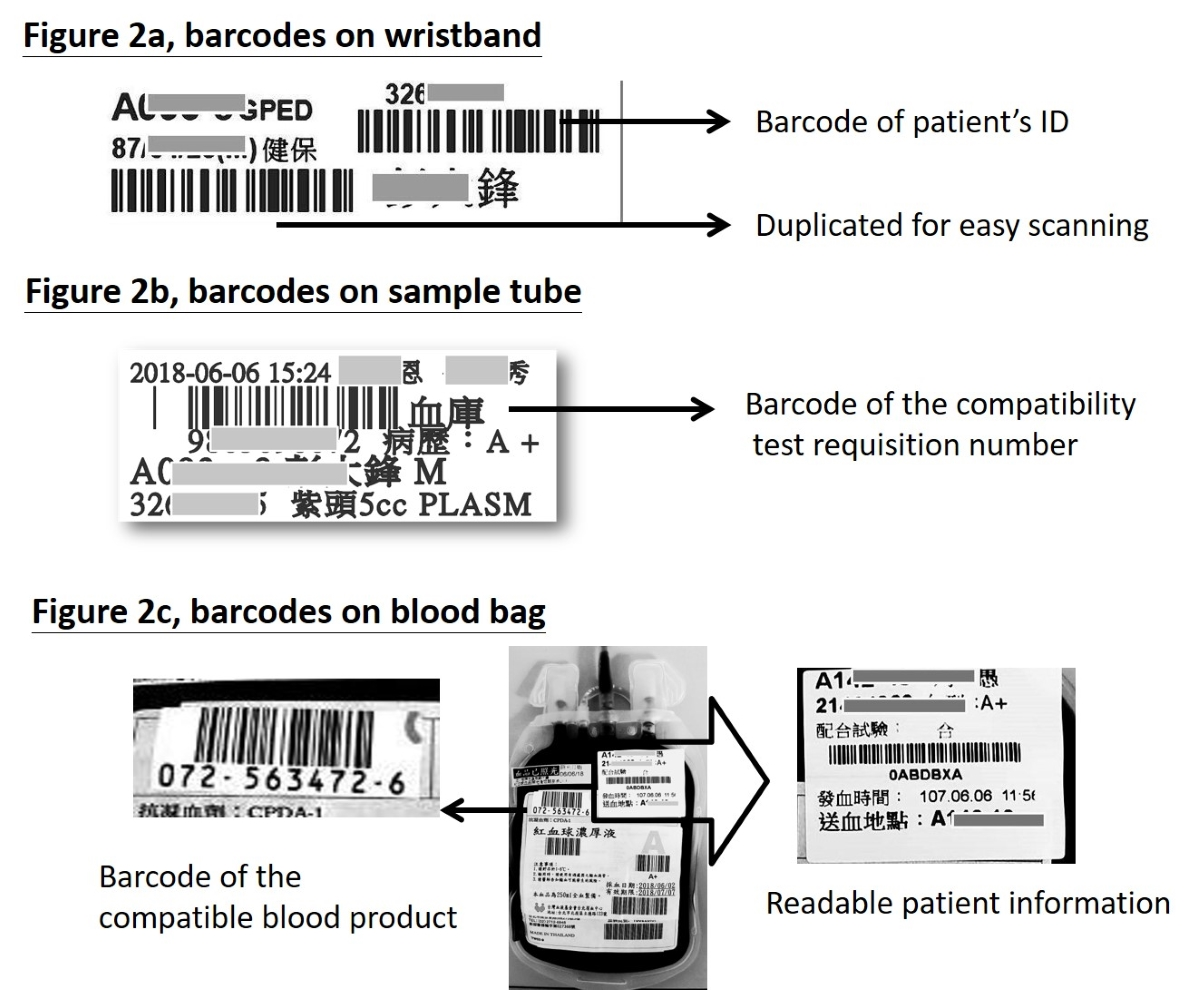
Barcodes used for the BCTM system. BCTM: bar code–based transfusion management; ID: identification.

A label printer and three drawers for different types of blood sampling tubes are added to the mobile nursing cart (Figure 1) to convert it to a phlebotomy cart for nurses to label sample tubes at bedside. The model of label printer we selected can print a 5 × 2 cm^2^ sticker. This size of the sticker is apt for easy sample tube labeling and allows sufficient readable information (such as patient’s name, bed number, the test requested, the name of staff performing the task, and the time of sampling) to be printed on it along with the specific barcode assigned by the HIS/LIS for the filled sample tube (Figure 2). With the alignment of the barcode systems of our laboratories, the filled sample tube can be directly put through the automated systems linked with the LIS, which also increases the efficiency of our laboratories. However, a paper requisition of the compatibility test printed at the nurse station was required to be wrapped around the labeled sample tube as a second source of information for verification and was maintained until June 2013 (Table 2).

#### Redesigning the End-to-End Tracking System

As our Blood Centre laboratory and Transfusion Medicine Department has been accredited by the College of American Pathologists since 2003, we did not change the practices and processes in the Blood Centre, but have instead worked with them to develop appropriate barcode systems and link necessary information from the Blood Bank computer system with the HIS/NIS/BCTM to accomplish the electronic tracking of blood products. After the compatibility test for a prescription of transfusion therapy, a specific barcode assigned by the BCTM for the compatible blood product bag is labeled for tracking, while the original process to label the readable information of the blood bag remains unchanged (Figure 2). With the updated BCTM process, onsite scanning of the recipient’s wristband barcode and the blood bag barcode prompts the BCTM system to automatically check if the blood product bag is correct for the patient, while the nurse still needs to verify that the readable information on the label is consistent with the information presented on the BCTM system. Two-person verification requires a second staff member to log in to the BCTM system and scan the patient ID barcode and the barcode on the blood bag for a second time. Once the verification is complete, the nurse then starts the transfusion and monitors the patient’s condition. All the patient’s reactions and the actions taken are recorded in the NIS as part of the nursing record for this therapy. At the end of the transfusion, the caring nurse has to confirm the completion of the blood product administration through the BCTM system. By activating this confirmation process, all relevant information recorded in the NIS during transfusion can be consolidated into the BCTM system to generate a transfusion record in order to accomplish an end-to-end tracking electronically. With this change, the need for a paper form of tracking was eliminated.

#### Step-by-Step Reminders

Training for staff on standard procedures has been a challenge in our hospital, as we have approximately 2500 nurses to cover more than 5000 transfusion monthly. Staff that are unfamiliar with the procedure (22%) or deviate from standard procedures (12%) were major reasons of errors in 2008-2010 (Table 1). To cope with the challenge, the presentation of the BCTM on the touch screen of the phlebotomy cart has been designed and arranged to guide the caring nurse step by step with the standard procedure. The nurse at the bedside just logs into the BCTM system and activates the scanner to obtain patient ID; the BCTM system then will automatically present on the screen the most updated physician’s order for compatibility tests or transfusion therapy for that particular patient. Each next step of the standardized procedures will pop up automatically to prompt the caring staff to follow along with photos of the right type of sample tubes or the presentation of blood product bags. With these operations occurring at the bedside and the reminders given by the BCTM system, the nurses can focus more on the patient and services with fewer chances to be interrupted.

### Study of the Intervention

In the study hospital, the Transfusion Safety Committee (TSC), which consists of representatives from the Nursing Department, Blood Centre, Clinical Laboratory, and Transfusion Medicine Department, governs transfusion safety and quality. The TSC meets quarterly to review errors (or near-miss incidents including incorrect labeling detected upon receipt by the Blood Centre, WBIT identified from the patient’s historical record in the HIS, or WBIT after resampling and rechecking by the Blood Centre if the first grouping result is not consistent with the patient’s own statement of blood type) and incorrect transfusion case reports presented by the Blood Centre and directs quality improvement actions. After a 3-month pilot run of the proposed BCTM system in two 40-bed wards to test its feasibility and to collect feedback from nurses to fine tune the process, the TSC approved a stepwise deployment of the BCTM system with the updated procedures and the use of phlebotomy carts, starting from regular wards and intensive care units in January 2011. The TSC granted the implementation of the BCTM into operation rooms in 2015 and emergency services in 2016.

**Figure 3:**
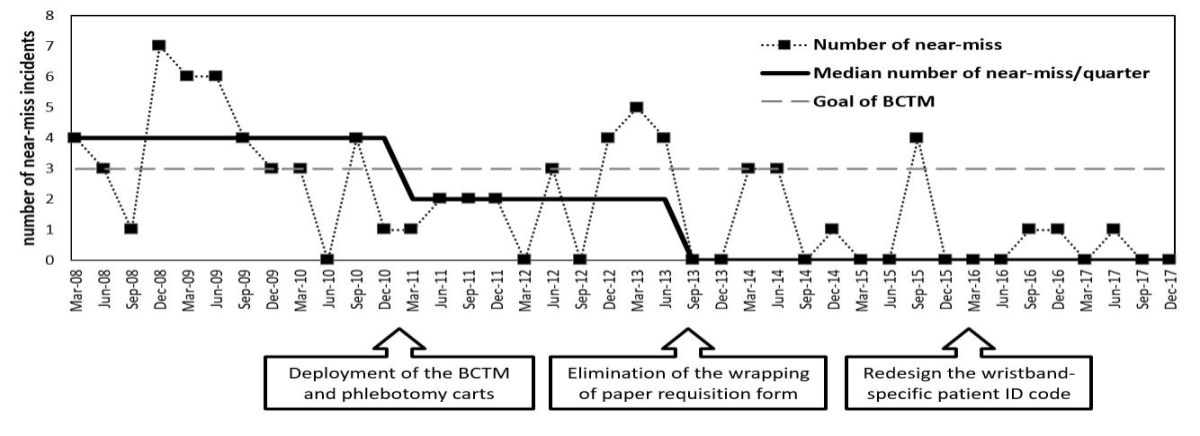
Run chart of near-miss incidents by quarter. BCTM: bar code–based transfusion management; ID: identification.

The project team reports the progress and quality indicator changes of the implementation of the BCTM system to the TSC. The number of occurrences of near-miss incidents during each quarter is plotted on a run chart by the BCTM project team to describe the progress of quality changes after interventions. An objective of reducing the number of near-miss events to fewer than three incidents per quarter was set as the goal, and the median of four quarterly near-miss incidents (from January 2008 to December 2010) was the baseline before the introduction of the BCTM system (Figure 3). At each quarterly TSC meeting, the causes and types of near-miss incidents encountered during the past 3 months were reviewed, and the required changes were proposed and discussed for implementation.

### Measures

The work of the batch preparation of sample tubes using preprinted labels was released from night shift nurses due to on-site labeling (Table 2). Timely verification of physicians’ orders through BCTM reduces communication lags and the wastage of the earlier preparation for the recently cancelled prescriptions. The simplified procedure for patient identification, two-person verification, and blood product identification as well as the saving from the discontinuation of the double entries to the paper form of the transfusion record and the elimination of paper requisition wrapping made the transfusion practices more efficient. After training and implementation to wards, first-line nurses welcome the updated procedures.

### Statistical Analysis

Incident reports of near-miss cases from January 1, 2008, to December 31, 2017, were retrieved from TSC quarterly meeting records and were reviewed and categorized by the authors of this report. The numbers of prescriptions for blood type matching by year from 2008 to 2017 were retrieved from the HIS for the annual error rate calculation. The number of occurrences of near-miss events by year from 2011 to 2017 was compared to the mean number of annual occurrences of near-miss incidents in 2008-2010, to examine if the introduction of the BCTM system in 2011 could have reduced errors. The number of occurrences of near-miss events by year from 2014 to 2017 was also compared to the mean annual occurrence of near-miss incidents in 2011-2013 to reveal the impact of the discontinuation of wrapping paper requisition forms around the labeled sample tubes after June 2013. Poisson statistics in Microsoft Excel (using the POISSON.DIST function. Version 2010. Redmond, WA: Microsoft Corp), assuming each occurrence was independent and rare (approximately 60,000 orders for blood matching were placed each year), was used to test if the error reduction brought by the BCTM and if the mentioned interventions were statistically significant (*P*<.05). The major outcome measurements of this study were to test if the BCTM system could effectively reduce the number of errors after its full implementation in the study hospital in 2016 and if the reduction could persist in 2017.

## Results

After introduction of the BCTM, the quarterly numbers of near-miss incidents met our objective to have less than three events per quarter, from quarter 1 in 2011 until quarter 3 in 2012 (Figure 3). A total of 13 incidents of mislabeling occurred in the fourth quarter of 2012 and the first half of 2013. Among these, four incidents involved incorrect paper requisitions wrapped around the tubes that had been sampled and labeled at the bedside. The four samples with incorrect paper requisition forms were verified by resampling to have the correct blood of the intended patient in the tube according to the label printed onsite. Based on the proven record of the BCTM system and the support of the Blood Centre, the TSC agreed that the need for paper requisition forms was redundant, and the process of wrapping paper requisition around the labeled sample was discontinued in June 2013.

With elimination of wrapping paper requisition around labeled tubes in June 2013, a median quarterly number of two near-miss events from January 2011 to June 2013 was set as an updated performance baseline (Figure 3). From September 2013 to the end of 2017, for a total of 18 quarters, there were 11 quarters (61%) with no near-miss incidents. The median number of near-miss incidents reached 0 per quarter and is set as the current performance standard. The two events of staff scanning patient ID barcodes from charts instead of scanning them from wristbands and four near-miss incidents that occurred in the third quarter of 2015 triggered the redesign of the wristband-specific patient ID barcode to ensure compliance to scanning wristband ID barcode for patient verification. The number of near-miss incidents stabilized at 0 to 1 per quarter in 2016 and 2017 (Figure 3).

The deployment of the BCTM system was performed stepwise, starting from the regular wards and intensive care units in 2011 to the operation rooms in 2015 and finally to the emergency services in 2016. Compared to the mean annual occurrence of 14 near-miss events in 2008-2010, the annual occurrence of near-miss events was significantly reduced after the introduction of BCTM (except in 2013 [*P*=.12]; Table 4). To examine the effectiveness of the discontinuation of paper requisitions wrapping in mid-2013, we use the mean annual occurrence of 7.46 from 2011 to 2013 as the baseline and found that the impact was significant when all the nursing units adopted BCTM in 2016 (*P*=.02) and the impact was sustained in 2017 (*P*=.004). After the implementation of the BTCM system in 2011, only one incorrect blood transfusion during an operation in 2016 was reported, due to a failure to scan the patient’s wristband ID barcode, which was covered by sterile drapes.

**Table 4:** The occurrence of near-miss incidents by year.

Type of error	Year									
	2008	2009	2010	2011	2012	2013	2014	2015	2016	2017
Identifier on sample tube and requisition not consistent	7	7	3	3	1	4	5	0	1	0
Identifier on sample tube incomplete or missed	5	6	2	2	3	2	1	3	0	1
Identifier for ABO testing and/or requisition not double verified	1	2	1	1	0	0	0	0	0	0
Inconsistency of the identifiers on the sample tube and ABO testing label	0	4	0	0	0	1	0	0	0	0
WBIT^a^	2	0	2	1	3	2	1	1	1	0
Total cases of wrong labeling and/or WBIT	15	19	8	7	7	9	7	4	2	1
Number of doctor’s orders	47,756	50,645	53,346	51,313	57,337	56,389	57,406	59,771	61,563	68,326
Annual error rates of incorrect labeling and/or WBIT (%)	0.03	0.04	0.02	0.01	0.01	0.02	0.01	0.01	0.002	0.001
Cumulative Poisson probability of near-miss occurrence^c^	—^b^	—	—	.03	.03	.109	.03	.002	<.001	<.001
Cumulative Poisson probability of near-miss occurrence^d^	—	—	—	—	—	—	.5	.12	.018	.004

^a^WBIT: wrong blood in tube with correct label.

^b^Not applicable.

^c^Based on the average occurrence in 2008-2010, mean=14.

^d^Based on the average occurrence in 2011-2013, mean=7.67.

## Discussion

With the full implementation of BCTM in 2016, the discontinuation of paper requisitions wrapping in 2013, and the introduction of wristband-specific patient ID barcode in 2015, the reduction in error occurrence in 2016 was statistically significant (*P*=.02) and sustained in 2017 (*P*=.004), using 2011-2013 as baseline. The objective of BCTM to reduce the near-miss rate to fewer than three incidents per quarter was achieved and sustained, as the occurrence of near-miss events decreased and stabilized at 0 to 1 per quarter for 8 consecutive quarters from quarter 1 in 2016 to quarter 4 in 2017 (Figure 3). There was no incident of WBIT and no incorrect blood transfusions in 2017, when a total of 164,495 bags of blood components were given to patients. Compared to the aggregate rates of 7.4 instances of mislabeling (306 specimens) and 0.43 instances of WBIT (10/23234) per 1000 specimens reported in the College of American Pathologists Q-Probes study for the first quarter of 2015 [10], our achievement of 0.015 instances per 1000 specimens (1/68326) in 2017 was satisfactory (Table 4).

The near-miss events reported here were detected by our Blood Centre when receiving and testing the samples. These reports have not included the near-miss incidents intercepted before leaving the nursing stations; hence, an underestimation of near-miss events may have occurred, especially before implementation of the BCTM system. With the current BCTM system, the need to check the correctness of the label at the nursing stations before sending out the sample to the Blood Centre is reduced, as only one label is used on site, providing fewer opportunities for errors and less possibilities of underreporting.

The implementation of BCTM addresses most of the major challenges identified by the root cause analysis of the 41 near-miss incidents that occurred in 2008-2010 (Table 1). The near-miss events before BCTM that were caused by staff being interrupted by other urgent issues in an open environment (41%) reduced by moving batch preparation of sample tubes at the nursing station to bedside labeling. BCTM simplifies labeling procedures and tackles 14% of near-misses that were related to complicated labeling procedures. The situation of staff being unfamiliar with the procedure (22%) or deviations (12%) from standard procedures is corrected by the step-by-step reminders showing on BCTM screens. Although the lack of staff members (10%) for two-person verification is still an issue for small ward units, we are working with our TSC to adopt the most updated National Patient Safety Goals, effective from January 2019 (NPSG.01.03.01), recommended by the Joint Commission Resource, to modify the current two-person verification process to a one-person verification process accompanied by an automated identification technology such as bar coding [15].

The deployment of nursing carts and phlebotomy carts enable us to deliver patient-centered care at bedside. Although it is difficult to provide a cost-effectiveness estimation of our investment to the BCTM, we believe the monetary cost is considerably less than that estimated in the past [16]. With a hospital-wide wireless environment and in-house IT support, the cost to build a BCTM system is shared with other barcode–based systems such as BCMA and laboratory systems for patient specimen collection [17]. For instance, in the study hospital, we have approximately 100 phlebotomy carts, two carts to cover a 40-bed unit, each costing approximately US $2500, to handle approximately 1,350,000 requisitions for blood sampling (including blood typing) each year. Assuming a simple 5-year depreciation of the cost of a phlebotomy cart, the shared cost of a phlebotomy cart for each blood sampling activity is approximately US$ 0.037 (US $2500 × 100 carts × 0.2)/1.35 million samples, which can be easily covered by the savings in error prevention and the improved efficiency.

The surge of near-miss incidents in quarter 4 of 2012 and the first half of 2013 (Figure 3) triggered the discontinuation of wrapping paper requisition around the labeled sample tube in June 2013. Five cases of sample tubes wrapped with the incorrect paper requisitions still occurred in early 2014 (Table 4); these were caused by slow adoption of the new process in some units and the stepwise implementation of the BCTM system in the study hospital. After the full deployment of BCTM, no such error occurred in 2017. This demonstrated that a leaner process based on a reliable mechanism can reduce errors caused by conflicting information generated from duplicated procedures [14].

“Workarounds” in the BCMA, that is, staff members scanning barcodes that contain patient ID information from the working environment but not from the wristband of the patient [18], were also observed in two of the four near-miss incidents reviewed by the TSC in September 2015. To remedy this “workaround,” the Information Department of the study hospital redesigned the barcode system to incorporate the time of printing into the ID barcode of the wristband, which can only be printed at designated printers. Starting in December 2015, our HIS and all subsystems only accept the most recently printed wristband barcode ID for patient identification. It is worth mentioning that the study hospital also empowers patients to protect their own safety [19,20] by explaining the importance of the ID barcodes on the wristband at admission and asks patients to remind staff to check their wristband as part of positive patient identification, should the staff member fail to do so.

According to the log of the nursing practice in the NIS, the compliance with the barcode scanning of patients’ wristbands reached 97% in 2017 (data on file). There were still circumstances that caused staff members to bypass the electronic system for urgent management. Standard procedures of paper-based blood sampling and transfusion management systems are still effective in our hospital, but are reserved for system failures or other urgent situations.

Although we observed the initial success of our BCTM from quarter 1 in 2011 until quarter 3 in 2012, when the objective to have less than three events per quarter was reached, the initial reduction in errors was not sustained (Figure 3), as we had increased cases of wrong wrapping of paper requisitions and “workaround” incidents. These reflect that our staff might have adjusted their practice to balance patient safety in the context of fluctuating demands and challenging work environments and equipment [21]. This might also explain why some studies report the usefulness of barcode–based systems on prevention of medical error [22], but are not supported by real-world situations [9,10]. From our experience, we believe the continuous PDSA efforts led by the Nursing Department, the quarterly review with the TSC to upgrade the system, and the empowerment of patients to support wristband-specific ID barcode scanning are the most critical success factors for a significant reduction in errors in 2016 and 2017.

Nevertheless, it is interesting to note that in 2017, a filled sample tube with no label on it was received by the Blood Centre and it was later found that the printed sticker was still left in the printer on the phlebotomy cart. This case shows that human errors still occur on occasions. The need for full attention from caring staff cannot be totally replaced by a computer-assisted system. We are still monitoring the trend and conducting quarterly review meetings with our TSC to ensure transfusion safety.

## Abbreviations

BCMA: Bar Code Medication Administration
BCTM: Bar Code based Transfusion Management
COPE: Computerized Physician Order Entry
HIS: hospital information system
IBM: International Business Machines
ID: unique patient identification
IT: information technology
LIS: Laboratory Information System
NIS: Nursing Information System
PDSA: Plan-Do-Study-Act
SHOT: Serious Hazards of Transfusion
SQUIRE 2.0: Standards for QUality Improvement Reporting Excellence
TSC: Transfusion Safety Committee
WBIT: wrong blood in tube
